# Homozygosity for a hypomorphic mutation in frizzled class receptor 5 causes syndromic ocular coloboma with microcornea in humans

**DOI:** 10.1007/s00439-024-02712-y

**Published:** 2024-11-06

**Authors:** Vianney Cortés-González, Miguel Rodriguez-Morales, Paris Ataliotis, Claudine Mayer, Julie Plaisancié, Nicolas Chassaing, Hane Lee, Jean-Michel Rozet, Florencia Cavodeassi, Lucas Fares Taie

**Affiliations:** 1 Departamento de Genética, Asociación Para Evitar la Ceguera en México, Vicente García Torres No. 46 Barrio San Lucas, Coyoacán, Mexico City, C.P. 04030 Mexico; 2 Facultad de Medicina, Universidad Nacional Autónoma de México, Mexico City, Mexico; 3 School of Health and Medical Sciences, City St. George’s University of London, London, SW17 0RE UK; 4 Complex Systems and Translational Bioinformatics (CSTB), ICube Laboratory, UMR7357, University of Strasbourg, 1 rue Eugène Boeckel, Strasbourg, 67000 France; 5 Faculté des Sciences, Université Paris Cité, UFR Sciences du Vivant, Paris, 75013 France; 6 Laboratoire de Référence (LBMR) des Anomalies Malformatives de l’oeil, Institut Fédératif de Biologie (IFB), CHU Toulouse, Toulouse, France; 7 Centre de Référence des Affections Rares en Génétique Ophtalmologique, CARGO, site constitutif, CHU Toulouse, Toulouse, France; 8 3billion Inc., Seoul, South Korea; 9 Laboratory of Genetics in Ophthalmology (LGO), INSERM UMR1163, Institute of Genetic Diseases, Imagine and Paris Descartes University, Paris, 75015 France

## Abstract

Ocular coloboma (OC) is a congenital disorder caused by the incomplete closure of the embryonic ocular fissure. OC can present as a simple anomaly or, in more complex forms, be associated with additional ocular abnormalities. It can occur in isolation or as part of a broader syndrome, exhibiting considerable genetic heterogeneity. Diagnostic yield for OC remains below 30%, indicating the need for further genetic exploration. Mutations in the Wnt receptor *FZD5*, which is expressed throughout eye development, have been linked to both isolated and complex forms of coloboma. These mutations often result in a dominant-negative effect, where the mutated FZD5 protein disrupts WNT signaling by sequestering WNT ligands. Here, we describe a case of syndromic bilateral OC with additional features such as microcornea, bone developmental anomalies, and mild intellectual disability. Whole exome sequencing revealed a homozygous rare missense variant in *FZD5*. Consistent with a loss-of-function effect, overexpressing of *fzd5* mRNA harboring the missense variant in zebrafish embryos does not influence embryonic development, whereas overexpression of wild-type *fzd5* mRNA results in body axis duplications. However, in vitro TOPFlash assays revealed that the missense variant only caused partial loss-of-function, behaving as a hypomorphic mutation. We further showed that the mutant protein still localized to the cell membrane and maintained proper conformation when modeled in silico, suggesting that the impairment lies in signal transduction. This hypothesis is further supported by the fact that the variant affects a highly conserved amino acid known to be crucial for protein-protein interactions.

**Supplementary Information:**

The online version contains supplementary material available at 10.1007/s00439-024-02712-y.

## Introduction

Ocular coloboma (OC) is a congenital eye defect resulting from the incomplete closure of the embryonic ocular fissure between the 5th and 7th week of fetal life (Chang et al. 2006). OC varies in prevalence across populations, with rates ranging from 4 to 19 per 100,000 live births in Europe and higher rates in populations with increased consanguinity (Hornby et al. 2003; Shah et al. 2011). Globally, it stands as a significant cause of congenital blindness and visual impairment, contributing to approximately 11% of pediatric blindness cases (Chen et al. 2021). OC can affect one or both eyes and may impact one or more parts, including the optic disc, retina, choroid, ciliary body, lens zonules, iris, and eyelids (Leung and Ko 2020; Jiang et al. 2021). Microphthalmia, myopia and microcornea often feature with OC, forming part of the developmental eye anomalies spectrum known as Microphthalmia, Anophthalmia, and Coloboma (MAC) (Onwochei et al. 2000; Chen et al. 2021). Both isolated and complex OC can be part of a syndrome with systemic features (Lingam et al. 2021). OC can be caused by genetic mutations, which may be inherited in an autosomal dominant (AD), autosomal recessive (AR), or X-linked manner, and/or by environmental factors (George et al. 2020). OC appears to be genetically diverse, with only a few genetic loci consistently observed across unrelated families, and recent studies indicate that over 70% of cases still lack a definitive genetic diagnosis. (Rainger et al. 2017; Trejo-Reveles et al. 2023).

**Fig. 1:**
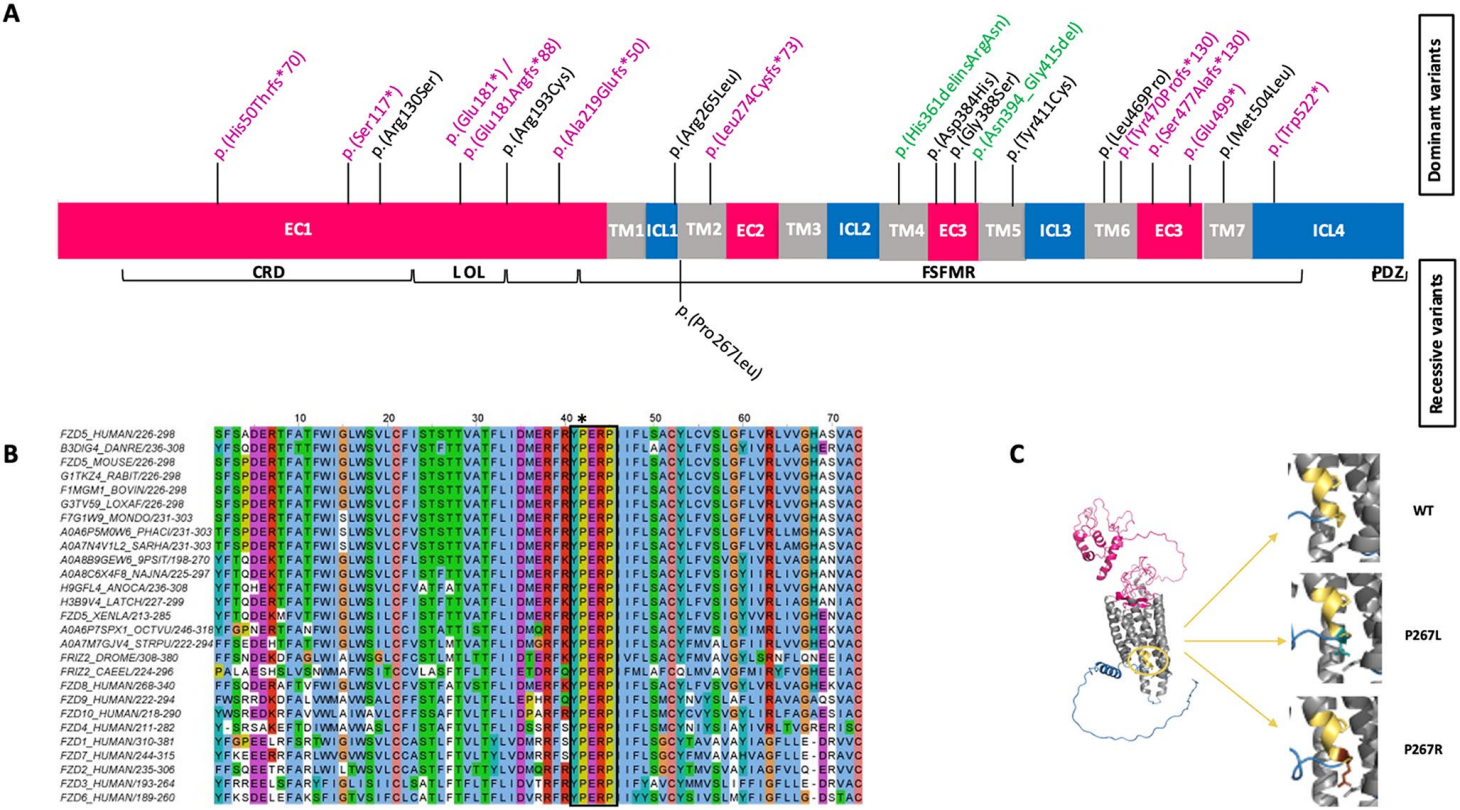
A novel recessive mutation in *FZD5*. (**A**) Likely pathogenic variants identified in *FZD5*. Truncating, missense, and in-frame variants are highlighted in violet, black, and green respectively. The upper section illustrates the position of the dominant (monoallelic) mutations identified in previous reports, while the lower section depicts the novel recessive (biallelic) mutation described in this study. FZD5 contains several important domains: a signal sequence at the N-terminus from amino acids (aa) 1–30; a conserved extracellular cysteine-rich Wnt-binding domain (CRD) from aa 31–150; a linker (**L**) from aa 151–193; an ordered loop (OL) from aa 194–227; and a Frizzled/Smoothened family membrane region (FSFMR) that includes seven transmembrane Frizzled domains (TM1: 228–260, TM2: 265–297, TM3: 314–345, TM4: 349–379, TM5: 396–428, TM6: 441–479, TM7: 499–522). A PDZ motif is located near the C-terminal at aa 583–585. (EC) extracellular domains and (C) cytoplasmic regions. (**B**) A multiple alignment was performed for 18 FZD5 orthologs from selected model species, along with all human Frizzled sequences. The YPERPI motif is highlighted (black rectangle); the p.Pro267Leu variant is indicated with an asterisk. Each sequence is identified by its UniProt accession number and the positions of its first and last amino acids in the selection. Amino acids color-coding follows the Clustal scheme. According to the alignment numbering, TM1 and TM2 comprise residues 3–34 and 42–72, respectively. Names of species are the following: DANRE, *Danio rerio* (Zebrafish); LOXAF, *Loxodonta africana* (African elephant); MONDO, *Monodelphis domestica* (Gray short-tailed opossum); PHACI, *Phascolarctos cinereus* (Koala); SARHA, *Sarcophilus harrisii* (Tasmanian devil); 9PSIT, *Amazona collaria* (yellow-billed parrot); NAJNA *Naja naja* (Indian cobra); ANOCA, *Anolis carolinensis* (Green anole, American chameleon); LATCH, *Latimeria chalumnae* (Coelacanth); XENLA, *Xenopus laevis* (African clawed frog); OCTVU, *Octopus vulgaris* (common octopus); STRPU, *Strongylocentrotus purpuratus* (Purple sea urchin); DROME, *Drosophila melanogaster* (Fruit fly); CAEEL, *Caenorhabditis elegans.* (**C**) (Left panel) Alphafold2 model of full-length human FZD5. The color code matches Fig. 1A. The YPERPI motif is highlighted and colored in yellow-orange. (Right panel) Zooming in on position 267 for the wild type (top), the P267L variant (middle), and the P267R variant (bottom). The two prolines of the YPERPI motif are depicted in yellow-orange sticks (P267 and P270), the leucine in deep teal sticks, and the arginine in chocolate sticks. Surrounding residues at position 267, including T339 from helix TM2, N350, and I353 from helix TM3, are shown in grey sticks

*FZD5* (OMIM* 601723) encodes a WNT receptor which displays an exclusive eye-specific expression pattern among FZD receptors across various stages of eye morphogenesis (Liu and Nathans 2008; Nikaido et al. 2013; Fujimura 2016). FZD5, like other FZD family members, features an amino-terminal cysteine-rich domain (CRD) facilitating WNT binding, seven-transmembrane domains (TM), and a cytoplasmic tail with a PDZ domain-binding motif at the carboxy-terminus (Fig. 1A).

Twenty dominant *FZD5* mutations have been reported in 21 families, predominantly resulting in isolated coloboma and less frequently in complex ocular coloboma (Human Gene Mutation database version 2024.2; https://www.hgmd.cf.ac.uk/ac/index.php) (Fig. 1A). However, as of now, no genotype–phenotype correlations have been identified (Aubert-Mucca et al. 2021; Holt et al. 2022).

Here, we present a case of syndromic bilateral ocular coloboma with microcornea, bone developmental anomalies, and mild intellectual disability whose exome sequencing analysis identified homozygosity for an ultra-rare FZD5 missense variant (p.Pro267Leu). Consistent with a loss-of-function effect, we demonstrate that overexpressing the *fzd5* mRNA carrying the missense change in zebrafish embryos does not affect embryonic development, whereas overexpression of wild type *fzd5* mRNA causes body axis duplications. TOPFlash in vitro analyses demonstrated that the missense variant exhibits partial loss of function and behaves as a hypomorphic mutation. The hypomorphic nature of the missense variant form is not due to a destabilization or absence of the protein, which still localizes to the cell membrane and shows overall normal conformation when modelled in silico. Our favoured interpretation is that the partial loss-of-function of the missense variant results from loss of efficient signal transduction, since the change affects a strictly conserved amino acid previously shown to be essential for protein-protein interactions. Overall, these findings mark the first reported recessive *FZD5* disease-causal variant and expand the *FZD5* disease spectrum to include syndromic complex ocular coloboma.

## Materials and methods

### Family

The proband, born to non-consanguineous parents of Mexican origin, sought consultation at the age of 31 for whole-exome sequencing (WES)-based molecular diagnosis of ocular coloboma with microcornea at the Hospital de la Ceguera in Mexico City (APEC). The patient, her parents, brother, sister, and her daughter were included in the study for segregation analysis of the *FZD5* c.800 C > T, p.Pro267Leu missense variant. All studies were conducted in accordance with the Helsinki Declaration of 1964. Prior to the molecular study, all participants or their legal guardians provided signed informed consent forms, including an iconographic informed consent form for the collection and use of clinical photographs.

### Whole exome sequencing

Genomic DNA was extracted from peripheral venous blood using a QIAamp DNA kit (Qiagen, Victoria, Australia) according to the manufacturer’s protocol. Whole exome sequencing (WES) was performed at 3 billion, Inc (Seoul, South Korea) to capture all known coding regions of 19,433 known human genes using xGen Exome Research Panel v2 (Integrated DNA Technologies, Coralville, Iowa, USA). Sequencing was performed by Novaseq 6000 (Illumina, San Diego, CA, USA) as 150 bp paired-end reads. Sequencing data was analyzed using an internal bioinformatics pipeline ‘EVIDENCE’ (Seo et al. 2020). In brief, the FASTQ file was aligned to the Genome Reference Consortium Human Build 37 (GRCh37) and Revised Cambridge Reference Sequence (rCRS) of the mitochondrial genome using BWA-MEM, generating 109.83 mean depth-of-coverage within the 34,366,188 bases of the captured region. Approximately 98.7% of the targeted bases were covered to a depth of ≥ 20x. Variants were called using GATK v.3, annotated with Ensembl Variant Effect Predictor (VEP) and classified and filtered according to the American College of Medical Genetics and Genomics (ACMG) guideline (Richards et al. 2015). The final list of variants was manually reviewed by medical geneticists. Pathogenicity of each variant was further reviewed using VarSome (Kopanos et al. 2019).

### Sanger validation and segregation analysis

The confirmation of the presence of the highest candidate variant identified by WES, *FZD5* c.800C > T (p.Pro267Leu; NM_003468.4), in the proband’s DNA and its segregation analysis in the family were studied by PCR amplification and Sanger sequencing, using primers designed from intronic sequences flanking exon 2: Forward: 5’-GTCACACCCGCTCTACAACA-3’ and Reverse: 5’-AGGAAGACGATGGTGCACAG-3’ and the BigDye^®^ Terminator v3.1 on an ABI 3500XL Genetic Analyzer (Applied Biosystems, Thermo Fisher Scientific, Courtaboeuf, France) as described in Gerber et al. 2021(Gerber et al. 2021). Data were analyzed using the ABI Sequencing Analysis 6 Software.

### Clinical assessment of the Pro267Leu variant carriers

The proband, homozygous for the variant, underwent comprehensive ophthalmological and systemic clinical examinations. Additionally, family members carrying the variant in heterozygosity (proband’s father, mother, brother, sister, and daughter) underwent thorough ophthalmic evaluations.

### Multiple sequence alignment of FZD5 across species and other members of the FZD family

Eighteen orthologs of human FZD5 were retrieved using the OrthoInspector website (https://lbgi.fr/orthoinspector/), (Linard et al. 2011; Nevers et al. 2019). The nine other Frizzled sequences (FZD1-FZD10) were aligned using PipeAlign2 (https://lbgi.fr/pipealign/), (Plewniak 2003). The percentage identity matrix of the ten FZD sequences was calculated using ClustalX2. Figure 1B was created using Jalview (Waterhouse et al. 2009).

### Prediction of the protein stability change upon single variation

The cryoEM structure (PDB code 6WW2, chain R), the crystal structure of FZD4 (PDB code 6BD4, chain A), and the AlphaFold2 models of human FZD4 and FZD5, as well as zebrafish Fzd5, were submitted to the DynaMut2 (Rodrigues et al. 2021) and the PremPS web server (Chen et al. 2020). This analysis aimed to predict the protein stability change upon modification of proline to leucine and arginine (Pro267 in human FZD5, Pro251 in human FZD4, and Pro277 in zebrafish Fzd5) on the structure of FZD5.

### Molecular dynamics web server-based simulation of mutant and wildtype FZD5

The AlphaFold2 model of human FZD5 was submitted to the CABS-flex 2.0 web server for rapid simulations to assess the flexibility of the structure, with a particular focus on predicting the flexibility level of the first intracellular loop (ICL1) within the context of the full-length structure. Default parameter values from the web server were utilized. Figures were created using PyMOL Molecular Graphics System, Version 3.0 Schrödinger, LLC.

### Generation of *fzd5* missense and loss-of-function forms in zebrafish

The zebrafish c.830C > T (p.Pro277Leu; *mi-fzd5*) variant corresponding to the human c.800C > T (p.Pro267Leu) substitution and the c.39del (E13Efs*30) mutation modeling a complete *FZD5* loss-of-function (*lof-fzd5*) were introduced into the zebrafish cDNA through site-directed mutagenesis by high-fidelity PCR amplification (Phusion polymerase) using the pCS2-*fzd5* plasmid containing the wild-type (wt) zebrafish *fzd5* cDNA (*wt-fzd5*) (Cavodeassi et al. 2005) as template and primers specific to the 830C > T and c.39del mutations (Forward: 5’- AGCGCTTCAAATATCtAGAGCGGCCGATTAT-3’ and Reverse: 5’- ATAATCGGCCGCTCTaGATATTTGAAGCGCT-3 and Forward: 5’- ACCATGGAACCTCAGGGATGCACCTGG-3’ and Reverse: 5’- CTGAGGTTCCATGGTGAAATGATGCTCG-3’respectively). Following amplification, template DNA was eliminated by digestion with the methylase-sensitive DpnI restriction enzyme for 1 h at 37 °C. XL1-Blue competent bacteria (Agilent Technologies) were transformed using the digested products and plated on LB Agar supplemented with ampicillin (100 µg/mL). Mutant variants from single colony minipreps were validated through Sanger sequencing using primers as described previously (Forward: 5’- CCTAACTGTGCACTGCCTTG-3’ and Reverse: 5’-ACCCCAGTGACACAAACAGA-3’for the *mi-Fzd5* variant and Forward: 5’-ATTTAGGTGACACTATAG-3’ and Reverse: 5’-CTCTGGCCACTCAAACCCAT- for the *lof-Fzd5* variant).

### Zebrafish lines and husbandry

AB/*tupl* wildtype zebrafish strains were maintained and bred according to standard procedures (Aleström et al. 2020). All experiments conform to the guidelines from the European Community Directive and British legislation (Animal (Scientific Procedures) Act 1986) and were conducted under the authority of Project Licence **PP5056153** granted to Florencia Cavodeassi by the British Home Office authorities.

### Synthesis of mRNA, embryo microinjections and in situ hybridization

Capped *wt-fzd5*, *mi-fzd5* and *lof-fzd5* mRNAs were synthesized using SP6 mMessage Machine (Ambion), following manufacturer’s instructions. 20 and 40 picograms/embryo (pg/emb) were injected into one-cell stage fertilized embryos and allowed to develop at 30 °C until the end of gastrulation (10 h post-fertilisation, hpf), as previously described (Cavodeassi et al. 2005). Double axes were visualized using antisense RNA probes for mRNA detection of *rx3* (eye field marker) and *pax2.1* (midbrain marker), synthesized with RNA-polymerases (Promega) and DIG-labelled nucleotides (Roche) following manufacturer’s instructions. Whole-mount in situ hybridization was performed as previously described (Hernández-Bejarano et al. 2015, 2022). Embryos were mounted in glycerol and imaging was performed under a Leica stereomicroscope connected to an IDS digital camara operated by IDS Software Suite.

### Analysis of protein localization

Fzd5 protein localization was examined in zebrafish embryos using a *fzd5*-RFP C-terminal fusion. A *mi-fzd5*-RFP fusion was generated by subcloning of the EcoRI-BspEI fragment of *mi-fzd5* into EcoRI-BspEI-digested pCS2-*wt-fzd5-RFP*. pCS2-*wt-fzd5-RFP* and pCS2-*mi-fzd5-RFP* were used as templates to synthesize mRNA for injection as described above. 200 pg/emb were injected into one-cell stage fertilized embryos, allowed to develop at 30 °C until dome stage (4.5 hpf), and fixed overnight in 4% paraformaldehyde. Embryos were briefly washed in PBS + Triton 0.3% and incubated with phalloidin-FITC (at 0.5µM to detect subcortical actomyosin) and Hoechst (at 1 µg/ml, to detect DNA) in PBS + Triton 1%+DMSO 1% for 4 h at room temperature, briefly washed in PBS and mounted in 1% low-melt-point agarose for imaging.

Imaging was performed in a Nikon A1R inverted confocal microscope with a 40X dry lens and images were processed with Nikon NIS Elements C software.

### TopFlash assays

pCS2-*wt-fzd5-RFP* and pCS2-*mi-fzd5-RFP* were used as templates to generate myc-tagged forms for transfection, using the the NEB Q5 site-directed mutagenesis kit (E0554; forward primer AGCGAAGAAGATCTGTAGAACTATAGTGAGTCGTATTAC; reverse primer AATCAGTTTCTGTTCGAGGACATGTGATGAG).

HEK293 cells were maintained in DMEM with 10% FCS at 37 °C in a humidified atmosphere of 5% CO_2_. Transfections were carried out using Polyethylenimine as described (Longo et al. 2013). Cells for TOPFlash assays were plated in quadruplicate on 24-well plates and transfected with M50 Super 8X TOPFlash along with pRLTK (Promega) for normalisation. Activation of the Wnt-pathway was achieved by co-transfection of *lrp6* and *fzd* constructs as described (Hua et al. 2018). Cells were processed 48 h after transfection with the Dual-Luciferase^®^ Reporter Assay System (Promega). Luminescence readings were made with a GloMax^®^ Discover Microplate Reader and data analysed using Excel and JASP software (JASP Team, 2024) JASP (Version 0.17.3) on macOS 10.15.7.

## Results

### Genetic findings

**Fig. 2:**
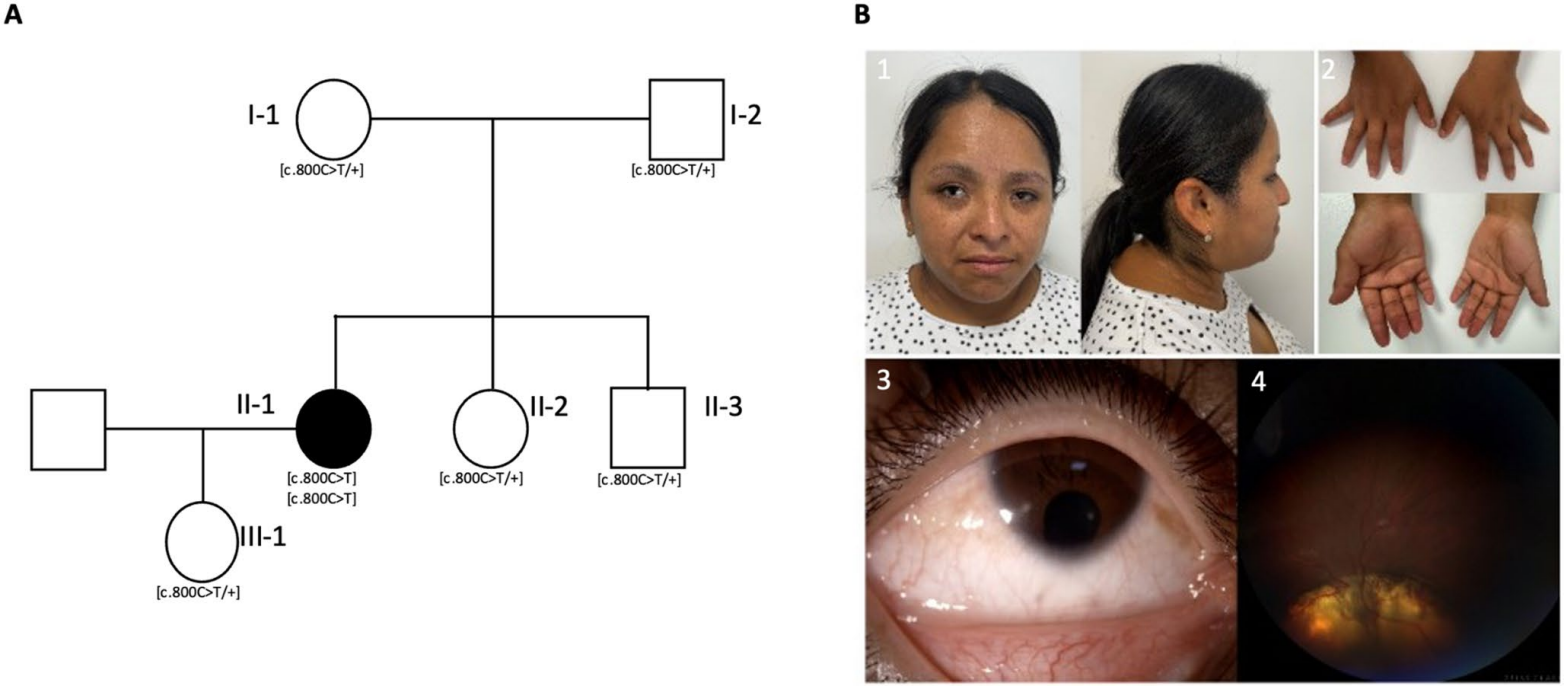
Clinical features associated with the c.800 C > T *FZD5* variant. (**A**)The family pedigree and segregation analysis reveal homozygosity for the NM_003468.4; c.800 C > T variant in the affected individual (II-1) and heterozygosity for the c.800 C > T variant in her parents (I-1 and I-2), siblings (II-2 and II-3), and daughter (III-1). (**B**) Photograph of the proband (1–4). (1) Demonstrated brachycephaly, facial asymmetry with freckles, strabismus, telecanthus, and epicanthus. (2) Hands exhibiting brachydactyly and slender fingers. (3) anterior segment of the right eye with iris coloboma and microcornea. (4) fundoscopy of the right eye demonstrated retinal and optic nerve coloboma

Analysis of the WES dataset from the proband revealed the sole convincing candidate variant to be a homozygous c.800 C > T (p.Pro267Leu; NM_003468.4; Fig. 1A) variant in exon 2 of the *FZD5* gene, encoding frizzled class receptor 5. Familial segregation analysis via Sanger sequencing confirmed biparental transmission, revealing both parents to be single heterozygous carriers for the variant despite the absence of known parental consanguinity. Additionally, the proband’s brother, sister, and daughter were identified as single heterozygous carriers (Fig. 2A).

The variation affects a highly conserved nucleotide (phyloP100: 10.003; CADD score of 32) and impacts an evolutionarily conserved amino acid within the strictly conserved YPERPI motif of the receptor, located at the boundary of IL1 and TM2. This motif is essential for regulating FZD-DVL interactions and may also contribute to receptor activation (Strakova et al. 2017). This region is preserved across many species and among all 10 FZD family members (FZD1–10; Fig. 1B).

The variant is absent from the gnomAD database but is referenced in ClinVar (ID: 2024628) as a variant of uncertain significance. However, according to the ACMG guidelines and Varsome analysis, which integrates multiple in silico predictors, the variant has a strong pathogenicity score (PP3). Further supporting its pathogenicity, this missense change is classified as likely pathogenic (score 0.994) using the recently developed AlphaMissense machine learning tool (Cheng et al. 2023).

### Clinical evaluation

The proband, a 31 year-old woman, reported a chief complaint of left eye (OS) deviation and nystagmus since the age of 2. Ophthalmic assessment revealed a best corrected visual acuity (BCVA) of 0.8 and 0.9 (LogMAR) in the right (OD) and left eyes, respectively. Refractive errors were noted as + 2 = -5 × 175° in the OD and + 1 = -5 × 175° in the OS. Anterior segment examination revealed bilateral iris coloboma, microcornea measuring 7.9 mm in the OD and 7.5 mm in the OS (corneal diameters: ± standard deviation of 11.71 ± 0.42 mm horizontally (Rüfer et al. 2005); narrow anterior chamber and narrow angle in both eyes (OU). Fundoscopy demonstrated retinal and optic nerve coloboma in OU (Fig. 2B). At the age of 3 years, B-scan ocular ultrasonography revealed an anteroposterior axial length of 21 mm in OU (mean axial length at 2 to 3 years of age is 21.4 ± 0.1 mm according to Gordon and Donzis; Gordon and Donzis 1985). Systemic examination revealed short stature (SD z-3.35), brachycephaly, facial asymmetry, telecanthus, epicanthus, and brachydactyly (Fig. 2B). Additionally, the proband exhibited mild intellectual disability, as documented by the WISC-R Wechsler test, with a score of 55 points. There was no reported family history of ophthalmic disorders or parental consanguinity.

Given the lack of reported recessive *FZD5* mutations to date, the proband’s relatives carrying the p.Pro267Leu variant in single heterozygosity (Fig. 2A) underwent an ophthalmological examination despite absence of overt symptoms. None of them exhibited any visible signs of ocular developmental anomalies, further supporting the recessive nature of the substitution.

### Predicting the impact of the Pro267Leu variant on protein stability and dynamics

**Fig. 3:**
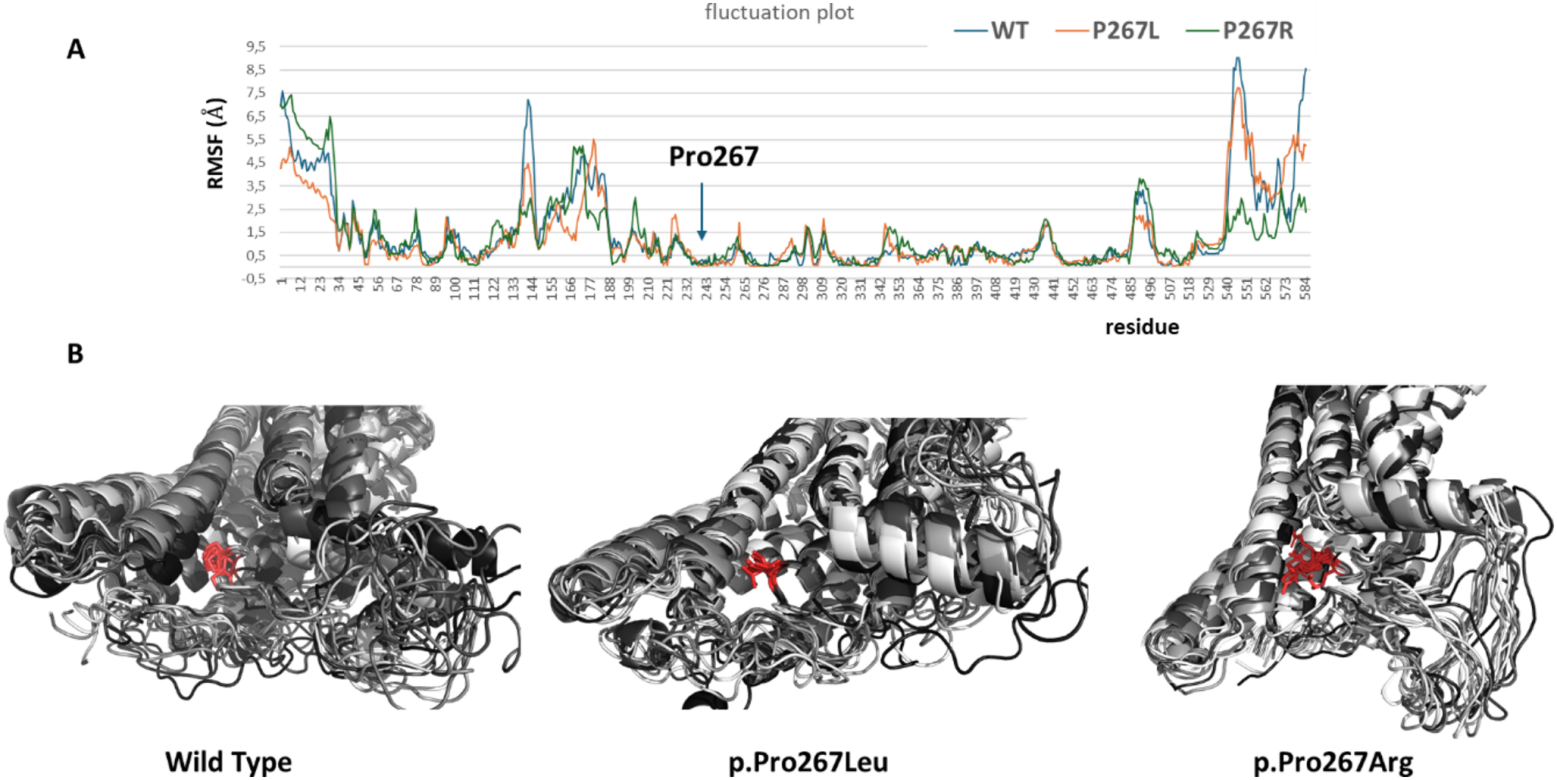
Fluctuation plot and molecular dynamics simulations (**A**) Fluctuation plot showing the residue fluctuation profile (RMSF) recorded throughout the simulation after global superposition. (**B**) Molecular dynamics simulations of the wild-type human FZD5, along with the Pro267Leu and Pro267Arg variants, are shown. Ten representative models from the simulation are depicted in cartoon form, with proline, leucine, and arginine illustrated as red sticks

The identification of a heterozygous dominant FZD4 variant (p.Pro251Arg) affecting the same conserved amino acid as in FZD5 in our patient (p.Pro267Leu) raises questions about whether Arginine could exert a different effect compared to Leucine on the final activity of the protein. One possible explanation might be a dominant effect of the p.Pro251Arg variant and a loss-of-function effect of the p.Pro267Leu substitution. Thus, we investigated the potential effects of substituting proline with leucine and proline with arginine at positions Pro267 in human FZD5 (Fig. 1C), Pro251 in human FZD4, and Pro277 in zebrafish Fzd5 (Fig. 1B) using experimental structures and AlphaFold2 models (Table 1). Pro267 corresponds to the first proline of the highly conserved YPERPI motif found within FZD5 orthologs and all Frizzled family members, marking the beginning of TM2 (Fig. 1B). Predictive tools DynaMut2 and PremPS suggest that substituting proline with leucine slightly destabilizes the structures of all proteins considered. In contrast, substituting proline with arginine significantly destabilizes protein structure (ΔΔG > 1 kcal/mol), indicating that the amino acid’s nature, as well as its position in the structure, profoundly affects protein stability (Table 1). In addition, molecular dynamics simulations demonstrate that the region encompassing the end of TM1, ICL1, and the start of TM2 remains stable regardless of the amino acid type. While the side chain conformations of proline and leucine at position 267 are quite similar, arginine displays significant flexibility during the simulation. This flexibility could potentially destabilize interactions with partners such as DVL (Fig. 3).

**Table 1:** The values of ΔΔG in kcal/mol represent the free energy change resulting from the variation. The following abbreviations are used: 6WW2 refers to the PDB code of the cryoEM structure of human FZD5; 6BD4 refers to the crystal structure of human FZD4; AF2 refers to the AlphaFold2 model.The numbering of the relevant proline is indicated for each protein: P267 in human FZD5, P251 in human FZD4, and P277 in zebrafish FZD5. It is worth noting that a negative value for DynaMut2 and a positive value for PremPS suggest a potential destabilizing effect on the structure

Amino acid variation	DynaMut2					PremPS				
	hFZD5 P267		hFZD4 P251		zFZD5 P277	hFZD5 P267		hFZD4 P251		zFZD5 P277
	6WW2	AF2	6BD4	AF2	AF2	6WW2	AF2	6BD4	AF2	AF2
Pro to Leu	-0.2	-0.5	-0.6	-0.4	-0.5	0.7	0.7	1.5	0.5	0.8
Pro to Arg	-0.7	-1.2	-1.1	-1.2	-1.1	1.6	1.8	1.8	1.6	1.8

### Functional analysis indicates that the p.Pro267Leu variant in FZD5 is likely a hypomorphic mutation

In silico modelling and pathogenicity predictive tools suggest a profound effect of the Pro267Leu substitution on *FZD5* function. To assess whether this was the case we turned to an in vivo functional approach. Previous studies have shown that injecting mRNA encoding for a wild type form of *fzd5* (*wt-fzd5*) into one-cell stage zebrafish embryos results in a partial or complete body axis duplication, which can be detected at the end of gastrulation by analysing the expression of the eye field marker *rx3* and the midbrain marker *pax2.1* (Cavodeassi et al. 2005). Co-injection of *wt-fzd5* mRNA with morpholinos blocking *fzd5* translation restores normal development, confirming the specificity of the double axis phenotype (Cavodeassi et al. 2005). We reasoned that injection of mRNA encoding for the missense variant identified in this study would not lead to double axes if the variant resulted in a loss-of-function. To reproduce the p.Pro267Leu *FZD5* variant in the zebrafish, we identified the homologous amino acid (Pro277) and generated the corresponding mutation by site-directed mutagenesis. mRNA encoding for the resulting p.Pro277Leu zebrafish *fzd5* variant (missense *mi-fzd5*) was injected into one-cell stage zebrafish embryos and the effect on axis establishment compared to *wt-fzd5* injection. A frameshift at the start of the open reading frame was also generated as a negative, complete loss-of-function control (*lof-fzd5*; see materials and methods).

**Fig. 4:**
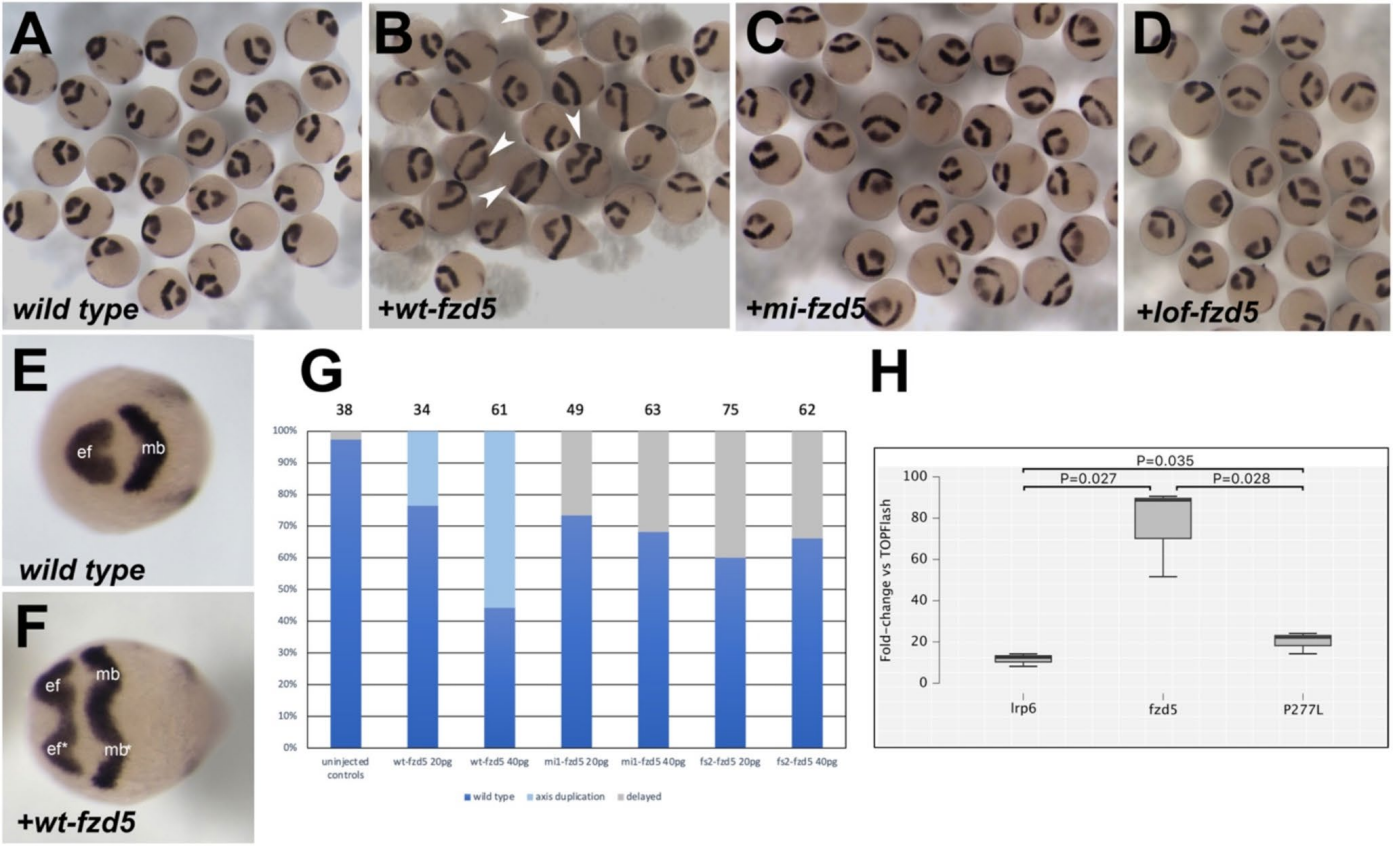
A missense variant in zebrafish Fzd5 mimicking the Pro267Leu variant does not induce a secondary neural axis. (**A**-**F**) Expression of *rx3* and *pax2.1* (dark purple) in 10hpf wild type embryos (**A**, **E**) and embryos injected with a *wt-fzd5* (**B**, **F**), *mi-fzd5* (**C**) or *lof-fzd5* (**D**) mRNA. Arrowheads in (**B**) highlight some of the embryos displaying double axes. (**E**-**F**) Partial or complete double axes are apparent by the duplication of the *rx3*-positive eye field domain (ef) and the medio-lateral expansion of the *pax2.1*-positive midbrain domain (mb). (**G**) Quantification of the effect of injections on body axis establishment. Total number of embryos analysed is displayed on top of the corresponding bars. (**H**) Fold change in luciferase activity of transfections of *lrp6*, *lrp6 + wt-fzd5* and *lrp6 + P277L-fzd5* normalised to activity of TOPFlash alone. Data pooled from three experiments with four replicas each

Injection of 20 and 40 pg/embryo of the *wt-fzd5* mRNA led to 23.5% and 55.7% of injected embryos displaying axis duplications, respectively (Fig. 4B, F, compare with wild type embryo phenotype in Fig. 4A, E; quantifications in Fig. 4G). In contrast, injection of the same amount of *mi-fzd5* and *lof-fzd5* mRNA resulted in no duplications (Fig. 4C-D, compare with wild type embryo phenotype in Fig. 4A; quantifications in Fig. 4G). A subset of *mi-fzd5* and *lof-fzd5* injected embryos displayed a mild developmental delay characterized by a broader neural plate (Fig. 4G), a delay that was rapidly recovered.

These results indicated that the ability of *mi-fzd5* to signal is severely compromised. To determine whether it retained any residual activity, we turned to a TOPFlash assay, a more sensitive in vitro approach. Previous reports indicate that the Wnt pathway is robustly activated by co-transfection of *fzd* receptors with *lrp6* (Hua et al. 2018). We generated C-terminal fusion constructs of both *wt-fzd5* and *mi-fzd5* with a *myc* epitope (see materials and methods), performed transfections of either *wt-fzd5-myc* or *mi-fzd5-myc* with *lrp6* and normalised the fold change in luciferase activity to the baseline obtained when transfecting the TOPFlash construct alone.

Co-transfection of TOPFlash with *lrp6* alone caused an 11.5-fold increase in luciferase activity. Addition of *wt-fzd5-myc* and *lrp6* resulted in an over 80-fold increase above the baseline while *mi-fzd5-myc* and *lrp6* together resulted in a 20-fold increase (Fig. 4H). Even though *mi-fzd5-myc* induces luciferase activity considerably below *wt-fzd5-myc*, this activity is significantly higher than transfection with *lrp6* alone, indicating that the missense variant retains some residual activity.

### The p.Pro277Leu substitution in zebrafish *fzd5* does not affect cell membrane protein localization

The results described above indicate that the *mi-fzd5* zebrafish form, reproducing the p.Pro267Leu *FZD5* human variant, behaves as a hypomorph. The in silico modelling presented above suggested a destabilization of the protein structure that may interfere with the efficient insertion of the protein into the plasma membrane. To analyze the subcellular localization of the mutant form, we generated C-terminal fusion constructs of both *wt-fzd5* and *mi-fzd5* with the red fluorescent protein (*wt-fzd5-FRP* and *mi-fzd5-RFP*, respectively; see materials and methods), synthesized mRNAs and injected them into one-cell-stage zebrafish embryos.

**Fig. 5:**
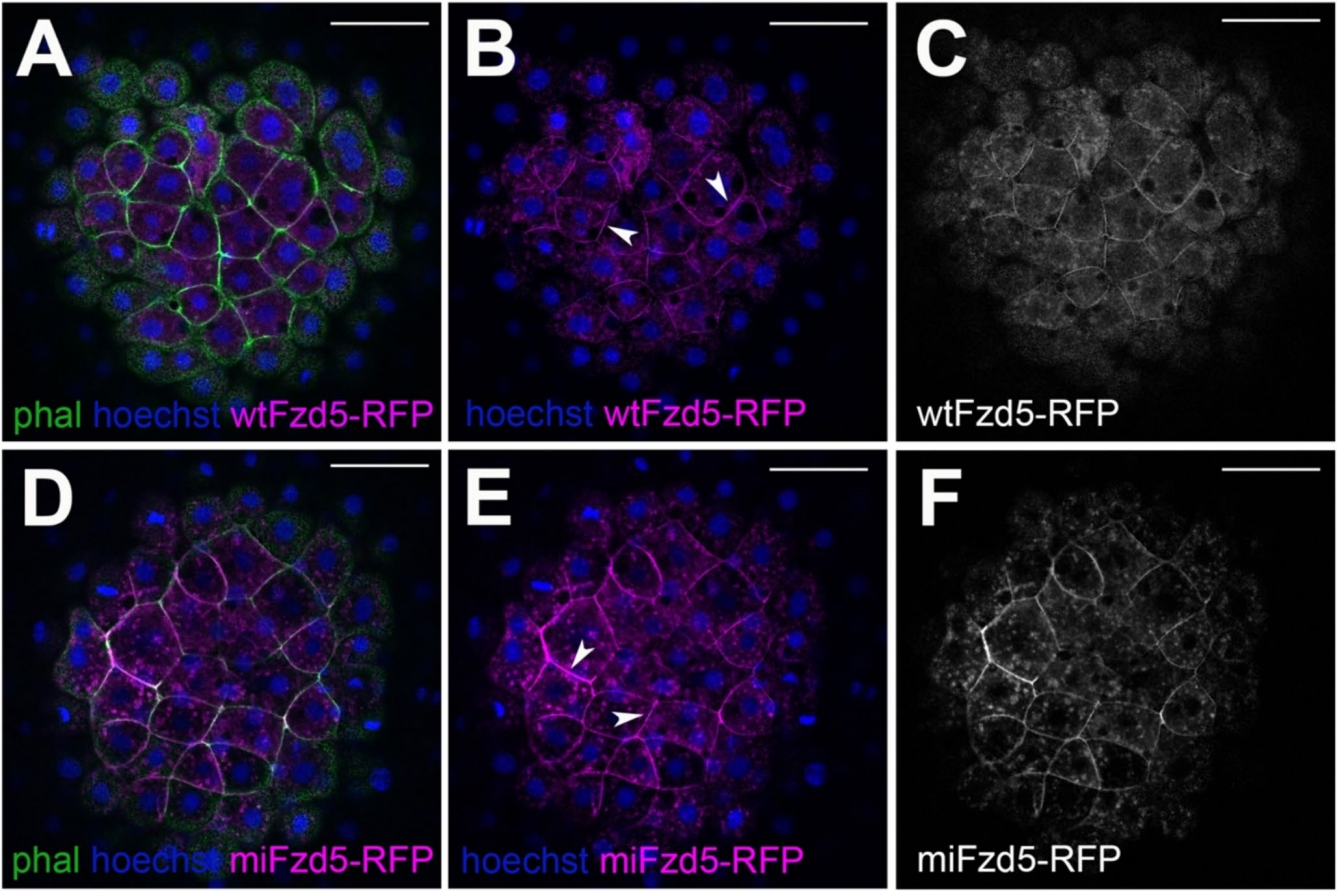
Subcellular localization of zebrafish Fzd5 is not affected by the Pro267Leu mutation. (**A**-**C**) Subcellular localization of wt-Fzd5-RFP (magenta, **A**-**B**; grey, **C**) and mi-Fzd5-RFP (magenta, **D**-**E**; grey **F**) in 4hpf embryos injected with the corresponding mRNA. Note the membrane localization of both wt-Fzd5-RFP and mi-Fzd5-RFP (arrowheads in **B**, **E**). Embryos were co-labelled with phalloidin-488 to reveal cell outlines (green) and Hoechst to reveal cell nuclei (blue). Scale: 50 μm

Both *wt-fzd5-RFP* and *mi-fzd5-RFP* localize to the plasma membrane in blastula-stage zebrafish embryos (Fig. 5 and Supplementary movies 1 and 2), indicating that the p.Pro277Leu substitution does not affect the ability of Fzd5 to traffic to the cell membrane and become inserted into it. Overall, these results indicate that the reduced activity of the p.Pro267Leu *FZD5* human variant is not due to lack of protein stability, and suggest that the mutant variant shows defective signal transduction upon interaction of FZD5 with WNT.

## Discussion

The association between *FZD5* and ocular anomalies has been extensively described (Aubert-Mucca et al. 2021; Holt et al. 2022). Heterozygosity for frameshift, nonsense and missense variants across various domains of *FZD5* have been involved in isolated or complex ocular coloboma. Moreover, incomplete penetrance with intrafamilial and intra-individual phenotypic variability has been observed (Aubert-Mucca et al. 2021). As there are no genotype-phenotype correlations, one possible explanation for understanding the variability in the clinical presentation could be that patients carry a combination of two heterozygous mutations in two genes of the WNT pathway, resulting in a synergistic effect due to digenic inheritance. Functional studies evaluating the impact of *FZD5* variants are sparse. Liu and colleagues proposed that the truncated variant p.Ala219Glufs*49, associated with isolated ocular coloboma, functions as a dominant-negative by sequestering WNTs and antagonizing WNT signalling (Liu et al. 2016). Additionally, Aubert-Mucca et al. suggested that some of the reported variants could also act as dominant-negative mutations (Aubert-Mucca et al. 2021). However, the mechanisms through which *FZD5* variants contribute to ocular anomalies remain elusive, necessitating further comprehensive functional studies.

The mechanism and mode of inheritance of *fzd5* mutations appear to differ in animal models. In mice, homozygosity for loss-of-function alleles has demonstrated early lethality due to defective angiogenesis in the placenta and yolk sac (Ishikawa et al. 2001). A conditional *fzd5* loss-of-function model in the eye has been reported to result in microphthalmia, coloboma, and persistent foetal vasculature (Liu and Nathans 2008). Studies in frogs (*Xenopus laevis*) and zebrafish (*Danio rerio*) have reported smaller eye primordia and reduced proliferation in the eye anlage of *fzd5*-morpholino-injected embryos (Cavodeassi et al. 2005; Liu et al. 2016).

In this study, we link iris, choroid, and optic nerve coloboma with bilateral microcornea, bone developmental anomalies, and intellectual disability to the biparental transmission of an ultra-rare *FZD5* missense variant in a Mexican individual, supporting the enhanced severity of recessive *FZD5* mutations.

Given the origins of both the proband’s parents from Mexico City and the extreme rarity of the variant, it is probable that it originated from a common ancestor, despite the absence of known consanguinity. The absence of ocular and systemic symptoms in the five familial relatives of the proband who carry the variant in single heterozygosity supports recessive inheritance. Further supporting the notion of a recessive *FZD5*-associated disease, overexpression of either the missense substitution (*mi-fzd5*) or a loss-of-function frameshift variant (*lof-fzd5*) in the zebrafish had minimal impact on embryonic development at the concentrations analyzed. In contrast, overexpression of the wild-type form (*wt-fzd5*) led to dose-dependent axis duplications. Of note, previous studies showed rescue with *fzd5* morpholino co-injection but not with a *fzd5*-mismatch morpholino, confirming the involvement of *fzd5* in the resulting phenotype. The same approach has been used in functional tests of other *fzd* variants in *Xenopus*, where overexpression of the wild-type form of *fzd* also leads to double axis phenotypes (Tauriello et al. 2012; Strakova et al. 2017). Despite the inability of *mi-fzd5* to induce double axes when injected in zebrafish embryos, this form still retains residual activity. This is apparent in TOPFlash assays where the level of luciferase activity, although strongly reduced when *mi-fz5* is transfected as compared to *wt-fzd5* transfections, is still higher than the level of luciferase activity induced by *lrp6* alone. Together, these findings indicate that the p.Pro277Leu *mi-fzd5* zebrafish variant, which mimics the human c.800 C > T p.Pro267Leu variant, results in the production of a defective Fzd5 protein with residual activity, and is consistent with the categorisation of this variant as a hypomorph. The hypomorphic nature of the variant likely explains the lack of phenotype in heterozygosis, and the recessive pattern of inheritance.

Extensive in vitro studies have been performed to systematically mutate specific amino acids in the FZD sequence and assess their functionality. These studies showed that critical residues in the three ICL and the C-terminal tail generate a binding pocket on the cytoplasmic surface of FZD receptors that facilitates the recruitment and docking of DVL to the plasma membrane upon pathway activation, an essential step in WNT signal transduction (Cong et al. 2004; Tauriello et al. 2012; Gammons et al. 2016; Strakova et al. 2017). FZD5 Pro267 and its homologous amino acid in other FZDs is a critical component of the described DVL binding domain, and its mutation severely affects DVL binding to FZD in vitro (Cong et al. 2004; Gammons et al. 2016). We propose the variant identified in this study generates a FZD5 protein unable to efficiently bind DVL and transduce WNT signalling.

FZD/DVL interactions activate both the canonical and non-canonical branches of the Wnt pathway, and FZD5 has been shown to activate both branches during eye development, in a highly context-dependent way (Van Raay et al. 2005; Cavodeassi et al. 2005). The TOPFlash luciferase assays presented in this report indicate that the p.Pro277Leu FZD5 variant is severely affected in its ability to activate the canonical branch of the Wnt pathway, but further experiments would be required to determine whether this variant has any effect on the ability of FZD5 to activate the non-canonical branch.

Frizzled (FZD) family proteins constitute a highly conserved group of cell surface receptors, including ten members (FZD1-10) in mice and humans. Notably, *FZD* genes linked to human diseases, such as *FZD2*, *FZD4*, and *FZD5*, typically exhibit autosomal dominant inheritance, with FZD6 being an exception with autosomal recessive transmission (Fröjmark et al. 2011; Saygı et al. 2019). Human FZD5 and FZD4 share significant sequence similarity in their 7TM domain, with approximately 62% identity. The identification of a heterozygous, dominant *FZD4* variant (p.Pro251Arg) in a patient with familial exudative vitreoretinopathy (FEVR) raises questions about how the FZD5 p.Pro267Leu variant, affecting the same conserved amino acid, leads to a recessive phenotype. One possible explanation lies in the nature of the substituted amino acids – a basic arginine in FZD4 compared to the hydrophobic leucine described here. These differences add further complexity to understanding the mode of transmission. Indeed, using predictive tools, we observed that replacing proline with leucine causes slight destabilization. This change could alter the shape of a highly charged (mostly basic) pocket adjacent to the cytoplasmic loop immediately following the YPERPI motif. Conversely, substitution of proline with arginine leads to significant destabilization, suggesting that the inherent properties of the amino acid, in addition to its specific location within the protein structure, have a substantial impact on protein stability. It has been suggested that certain mutated *FZD4* alleles could underlie recessive rather than dominant disease (Khan et al. 2017), and it would be interesting to determine whether these recessive variants have a less deleterious effect on protein stability. However, further investigations are required to establish a clear genotype/phenotype correlation.

Alongside the ocular phenotype, the proband also presented with intellectual disability. To our knowledge, this is the second individual harboring *FZD5* mutations associated with neurological anomalies, following the initial description by Aubert-Mucca and colleagues (Aubert-Mucca et al. 2021), who reported neurocognitive difficulties in a proband carrying the heterozygous c.1081_1082insGAA p.(His361delinsArgAsn) variant. Intellectual disability in these two cases may be related to defective FZD5 function in the hypothalamus, parafascicular nucleus of the thalamus and/or ventral telencephalon, where *FZD5* is expressed throughout embryogenesis (Shimogori et al. 2004; Burns et al. 2008; Liu and Nathans 2008; Nikaido et al. 2013). Finally, this marks the first report of an individual presenting with short stature, brachycephaly, facial asymmetry, telecanthus, epicanthus, and brachydactyly of the hands associated with *FZD5*. To date, *FZD5* expression has only been reported in the eyes and brain during embryonic development (Liu and Nathans 2008; Nikaido et al. 2013). Thus, determining whether these extraocular anomalies are caused by the *FZD5* mutation is challenging. However, it is noteworthy that several reports have highlighted the significant role of WNT signaling in regulating various aspects of skeletal development and maintenance (Liu et al. 2008, 2022; Hu et al. 2024). In our patient, WES analysis failed to identify any plausible gene responsible for the extraocular phenotypes. Further analysis is necessary to determine whether these associated phenotypes are unique to the recessive form of *FZD5* mutations. Additional studies will contribute to a more precise clinical characterization of conditions resulting from *FZD5* mutations and the pathogenic mechanisms of dominance, which, based on the absence of ocular anomalies in individuals carrying a recessive *FZD5* variant, likely exert a dominant-negative effect, leaving less than 50% of FZD5 activity.

In conclusion, here we report for the first time that biallelic variants in *FZD5* are responsible for syndromic bilateral ocular coloboma with microcornea. Our study suggests that both recessive and dominant mutations in *FZD5* contribute to ocular malformation, thus extending the clinical and genetic spectrum of *FZD5*-related patients. Additionally, the results presented here suggest that biallelic variants in *FZD5* could be responsible for syndromic forms of ocular colobomas. However, additional cases are necessary to clarify this scenario. Although no clear correlation could be established between the nature or localization of the variants within *FZD5* and the severity of the associated phenotype, the study presented here provides further evidence of the crucial role of the WNT pathway during eye development and organogenesis.

## Electronic supplementary material

Below is the link to the electronic supplementary material.

Supplementary Material 1

Supplementary Material 2

Supplementary Material 3

## Data Availability

The data supporting the findings of this study are available from the corresponding authors upon request.

## Declarations

### Rights retention statement

This research was funded in part, by the Wellcome Trust [Seed Award in Sciences 213928/Z/18/Z to FC]. For the purpose of open access, the authors have applied a CC BY public copyright licence to any Author Accepted Manuscript version arising from this submission.
