# Supplementary material for: Homozygosity for a hypomorphic mutation in frizzled class receptor 5 causes syndromic ocular coloboma with microcornea in humans
Source: Hum Genet. 2024 Nov 6;143(12):1509–21. doi: 10.1007/s00439-024-02712-y (PMC11576812; doi:10.1007/s00439-024-02712-y)
Supplement: Supplementary file 3 — Supplementary Material 3 [file 439_2024_2712_MOESM3_ESM.docx]

**Supplementary movie 1:** wt-Fzd5-RFP (red) localizes to the plasma membrane in a 4hpf zebrafish embryo. Confocal stack movie, 1.15 microns step.

**Supplementary movie 2:** mi-Fzd5-RFP (red) localizes to the plasma membrane in a 4hpf zebrafish embryos Confocal stack movie, 1.15 microns step.
